# A comparative study of fracture conductivity prediction using ensemble methods in the acid fracturing treatment in oil wells

**DOI:** 10.1038/s41598-023-50731-y

**Published:** 2024-01-05

**Authors:** Parsa Kharazi Esfahani, Mohammadreza Akbari, Yasin Khalili

**Affiliations:** 1 Department of Petroleum Engineering, Amirkabir University of Technology (Tehran Polytechnic), 424 Hafez Avenue, Box 15875-4413, Tehran, 1591634311 Iran; 2 Department of Mathematics and Computer Science, Amirkabir University of Technology (Tehran Polytechnic), 424 Hafez Avenue, Box 15875-4413, Tehran, 1591634311 Iran

**Keywords:** Computer science, Engineering

## Abstract

The study of acid fracture conductivity stands as a pivotal aspect of petroleum engineering, offering a well-established technique to amplify production rates in carbonate reservoirs. This research delves into the intricate dynamics influencing the conductivity of acid fractures, particularly under varying closure stresses and in diverse rock formations. The conductivity of acid fractures is intricately interconnected with the dissolution of rock, etching patterns on fracture surfaces, rock strength, and closure stress. To accurately predict fracture conductivity under different closure stresses, a robust model is necessary. This model involves assessing both the baseline fracture conductivity under zero closure stress and the rate of conductivity variation as closure stress fluctuates. Key among the influential factors affecting fracture conductivity is the type of rock within the reservoir. Understanding and predicting the behavior of different formations under disparate closure stresses poses a significant challenge, as does deciphering the diverse effects of treatment parameters such as acid injection rate and strength on fracture conductivity. In this study, the predictive power of XGBoost, a machine learning algorithm, was explored in assessing acid fracture conductivity in dolomite and limestone formations. The findings revealed XGBoost's ability to outperform previous studies in predicting fracture conductivity in both types of formations. Notably, it exhibited superior accuracy in forecasting fracture conductivity under varying treatment conditions, underscoring its robustness and versatility. The research underscores the pivotal role of closure stress, dissolution rate of rock (DREC), and rock strength in influencing fracture conductivity. By integrating these parameters into the design of acid fracturing operations, accurate predictions can be achieved, allowing for the optimization of treatment designs. This study illuminates the potential of XGBoost in optimizing acid fracturing treatments, ultimately bolstering well productivity in carbonate reservoirs. Furthermore, it advocates for the essential nature of separate modeling and analysis based on rock types to comprehend and optimize fracturing processes. The comparison between dolomite and limestone formations unveiled distinct conductivity behaviors, underlining the significance of tailored analyses based on rock type for precise operational optimization.

## Introduction

Due to the growing global demand for oil and gas, oil companies are facing increasing pressure to optimize their well production rates. In order to meet this demand, oil companies must focus on enhancing their production capabilities and improving the efficiency of their drilling and recovery processes. Acid fracturing is a well-stimulation technique used in the oil and gas industry to increase the productivity of a well^1^. It involves injecting acid into the formation at high pressure to create fractures or enlarge existing ones, which allows for better fluid flow and increased production. The acid used is typically hydrochloric acid or other types of acids that can dissolve minerals in the rock formation, creating channels for oil and gas to flow through. Acid fracturing is often used in carbonate formations, where traditional hydraulic fracturing may not be effective due to the low permeability of the rock. However, it can also be used in sandstone formations to improve production. Acid fracturing is considered a more environmentally friendly alternative to hydraulic fracturing because it uses less water and does not require the use of proppants^2^.

Acid fracture conductivity refers to the ability of an acid-treated fracture in a rock formation to allow fluid flow. When an acid is injected into a wellbore, it reacts with the rock formation and creates fractures or enlarges existing ones. These fractures provide pathways for oil, gas, or water to flow into the wellbore. The conductivity of these fractures is important because it determines how much fluid can flow through them^3^. A high conductivity means that more fluid can flow through the fracture, while a low conductivity means that less fluid can flow through. Factors that affect acid fracture conductivity include the type and concentration of acid used, the temperature and pressure of the reservoir, and the properties of the rock formation. To optimize fracture conductivity, engineers may use additives such as proppants or diverting agents to enhance fluid flow and prevent blockages^4^.

**Figure 1:**
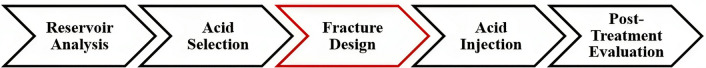
Steps for acid fracture design^3^.

Designing an acid fracturing treatment involves several steps and considerations. The following is a general overview of the process Fig. 1^5^:

1. Reservoir analysis: the first step in designing an acid fracturing treatment is to analyze the reservoir to determine its characteristics and properties. This includes analyzing the formation's lithology, permeability, porosity, and other geological features.
2. Acid selection: once the reservoir analysis is complete, the next step is to select the appropriate acid for the treatment. Factors to consider when selecting the acid include its reactivity, viscosity, and ability to penetrate the formation.
3. Fracture design: the next step is to design the fracture to be created in the formation. This involves determining the fracture length, width, and height necessary to achieve the desired production rate. The fracture design is typically based on the properties of the formation, the type of acid being used, and the desired level of conductivity.
4. Acid injection: once the fracture design is complete, the next step is to inject the acid into the formation. This is typically done using a high-pressure pump and specialized equipment designed to ensure that the acid is injected at the correct pressure and flow rate.
5. Post-treatment evaluation: after the acid has been injected into the formation, it is important to evaluate the effectiveness of the treatment. This involves monitoring the well's production rate, conductivity, and other factors over time to determine whether the treatment was successful^6–9^.

Fracture Design is the most important stage in determining Acid Fracture Conductivity. This is because the fracture design determines the size and shape of the created fracture, which directly affects the conductivity of the fracture. The design must take into account the properties of the formation, such as permeability and porosity, as well as the treatment parameters, such as acid volume, concentration, and injection rate. The goal is to create a fracture that maximizes the contact area between the reservoir and the wellbore, allowing for better fluid flow and increased production^10,11^.

To develop effective acid fracturing treatments, it is crucial to have a model that can simulate each stage of the process and predict the resulting fracture conductivity. Current industry practices rely on conductivity correlations derived from laboratory measurements of formations that are similar to those encountered in the field. While these correlations can inform the design of acid fracturing treatments, their effectiveness is limited by the variability in geological features and reservoir conditions between different formations. Therefore, improving the accuracy of prediction models may lead to more efficient and cost-effective fracturing treatments in the future^12^.

## Literature review

1. The Nierode and Kruk^13^ correlation for acid-fractured conductivity is a fundamental mathematical model used in the petroleum industry to estimate the conductivity of wells that have gone through hydraulic fracturing treatments with acid. This correlation is based on empirical data gathered from field experiments, which established a direct relationship between conductivity and the quantity of rock that was dissolved during the acid injection process, as well as the rock's hardness and embedment strength, which also impact conductivity. Nierode and Kruk^13^ correlation is a method that predicts conductivity at zero closing stress by taking into account the ideal fracture width, which is calculated by dividing the dissolved rock amount by the fracture area. This correlation describes the impact of closing stress on fracture conductivity, with exponential precision. However, this empirical model does not account for formation properties and, as such, is only suitable for predicting the lower end of the fracture conductivity range. Nonetheless, the Nierode and Kruk correlation represents an essential tool for the design and optimization of effective acid-fracturing treatments^13,14^.
  The conductivity of wells that have undergone hydraulic fracturing treatments with acid is strongly influenced by the formation characteristics, specifically, the rock hardness.
2. Nasr-El-Din et al.^15^ suggested modifying the Nierode and Kruk correlation, which had been lumped together, without taking into account the lithology. Through individual evaluations of data, by lithology, they recalculated the correndersonsonnndnlation constants, resulting in a more precise and differentiated set of correlations^15,16^.
3. Pournik et al.^17^ performed laboratory tests that were scaled to field conditions, and the results did not align with the predictions of the Nierode and Kruk correlation. Pournik concluded that the optimal acid system depends on closure stress and the treatment schedule, as the treatment can impact rock strength^17–20^.
  It was observed that there was an optimal contact time for each formation and acid, depending on closure stress levels, as high contact times could lead to a decline in conductivity rates, even at low closure stresses. Each formation responded differently to the same treatment, especially in the decline of conductivity rate with closure stress, as the strength of each formation was found to be unique^21,22^.
4. In a study by Motamedi-Ghahfarokhi et al.^23^, a robust intelligent model was developed using the Genetic Algorithm to estimate fracture conductivity based on various experimental data from different formations while considering the parameters and formation lithology. The study found that the formation lithology has a significant impact on the prediction of fracture conductivity^23^.
5. In another study by Akbari et al.^24^, artificial neural network models were developed to predict fracture conductivity accurately. The models incorporated experimental data from various formations and showed good agreement between predictions and data. The effects of rock type and treatment parameters on fracture conductivity were investigated and found to differ among formations under various closure stresses. Although an optimum point exists for achieving maximum fracture conductivity, it is difficult to determine because it varies by formation^24^.
6. In another study by Akbari et al.^25^, an intelligent model based on a genetic algorithm was developed to predict fracture conductivity accurately. The model incorporated experimental data from various formations and showed good agreement between predictions and data. Rock strength was found to significantly affect fracture conductivity under different closure stresses. However, at high closure stresses, predictive correlations became less precise due to the complexity of rock behavior. Therefore, fracture conductivity should be anticipated cautiously, particularly in soft formations, using various predictive correlations at high closure stresses^25^.

### Machine learning method compared to other methods for determining acid fracture conductivity

Machine learning has several advantages over analytical, numerical, empirical, and artificial intelligence methods in estimating fracture conductivity correlation.

1. Machine learning algorithms can handle large amounts of complex data and can automatically identify patterns and relationships in the data, which can be difficult or impossible for humans to detect using traditional methods. This makes machine learning particularly useful for estimating fracture conductivity correlations, which can be influenced by a wide range of geological and operational factors^26^.
2. Machine learning algorithms can adapt and improve over time as they are exposed to new data. This means that as more data becomes available, machine learning algorithms can refine their estimates and improve their accuracy.
3. Machine learning algorithms can handle non-linear relationships between variables, which is often the case in fracture conductivity correlations. This means that machine learning algorithms can provide more accurate estimates than traditional methods that assume linear relationships between variables^26^.
4. Machine learning algorithms can be used to develop predictive models that can be used to estimate fracture conductivity correlation in new wells or formations based on existing data. This can help to reduce the time and cost associated with conducting empirical tests and experiments^26^.
5. Another important point to consider is that finding the appropriate machine learning algorithm among the available options is crucial. Different machine learning algorithms have varying strengths and weaknesses, and selecting the most suitable algorithm for a specific problem can greatly impact the accuracy of the results. Therefore, it is important to carefully evaluate and compare different machine learning algorithms before selecting one for estimating fracture conductivity correlation. This can involve testing the algorithms on different datasets, evaluating their performance metrics, and considering factors such as computational efficiency and interpretability.

Overall, the use of machine learning algorithms has the potential to significantly improve the accuracy and efficiency of estimating fracture conductivity correlation in the oil and gas industry.

### Our study

In this study, the XGBoost machine learning algorithm, which is one of the most effective Ensemble Learning methods, was utilized. This study aimed to evaluate the performance of the XGBoost method for estimating acid fracture conductivity under different closure stress, DREC, and RES conditions, and to compare the results with previous studies to demonstrate that this approach can provide better estimates.

## Method of this study

### Ensemble learning

**Figure 2:**
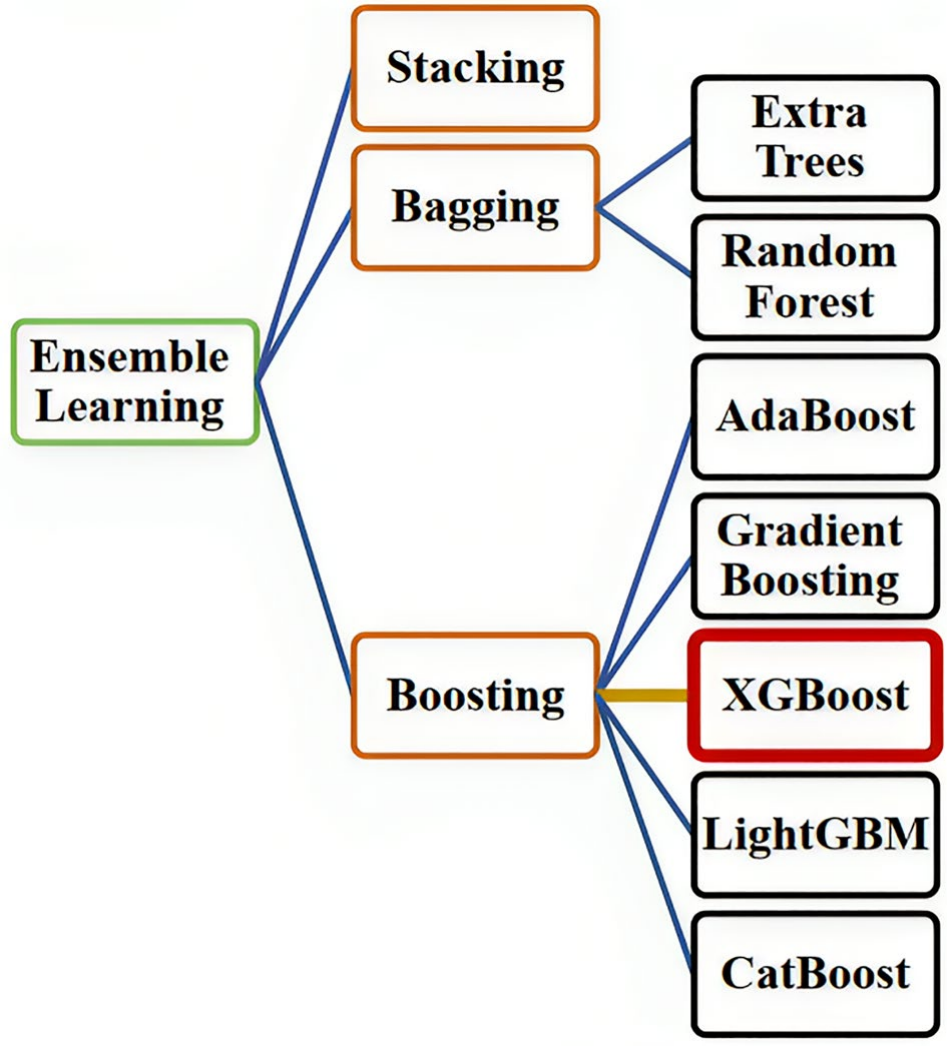
Various techniques used for ensemble learning^28^.

Ensemble learning is one of the most powerful methods which is used in this study due to its exclusivity. Ensemble learning is a machine learning technique that involves combining multiple models to enhance the accuracy and performance of prediction. It can be used in various machine-learning tasks like classification, regression, and clustering^27^. The fundamental concept of ensemble learning is to decrease the possibility of individual models making mistakes and improve the overall accuracy and resilience of predictions by combining multiple models. Bagging, boosting, and stacking are among the various techniques that can be used for ensemble learning Fig. 2^28,29^.

#### Bagging

Bagging is a method in which several copies of the original dataset are generated by random sampling with replacement. Each of these copies is then used to train a separate model. The predictions of these models are then combined to obtain the final prediction^8^. This method can be performed using the following techniques.

##### ExtraTrees

ExtraTrees is a variant of Random Forest that uses a randomized approach to selecting candidate splits for each node in the decision tree. Unlike Random Forest, which selects the best split based on the Gini impurity or entropy criterion, Extra-Trees randomly selects a set of candidate splits and chooses the best one based on a random threshold value. This randomization helps to decrease the variance of the model and enhance its generalization performance^30^.

##### Random forest

Random forest is an ensemble method that is based on bagging and combines multiple decision trees to create a more accurate and robust model. The technique involves randomly sampling the training data and features at each iteration to train a new decision tree. The final model is constructed by aggregating the predictions of all the individual trees. This approach helps to decrease the model's variance and prevent overfitting, making it a popular choice for various classification and regression tasks^30–32^.

#### Boosting

Boosting is an ensemble method that involves training multiple weak models sequentially on different subsets of the data, where each subsequent model is trained on the instances that were incorrectly classified by the previous models. The final prediction is obtained by aggregating the predictions of all these weak models^33^. This method can be performed using the following techniques.

##### AdaBoost

AdaBoost (Adaptive Boosting) is a popular boosting algorithm that works by training a sequence of weak classifiers on different subsets of the data, where each subsequent classifier focuses on the instances that were misclassified by the previous classifiers^33^.

##### Gradient boosting

Gradient boosting is an extension of AdaBoost that uses gradient descent optimization to iteratively train weak classifiers. This technique works by minimizing a loss function with respect to the model parameters and can be applied to both regression and classification tasks^33^.

##### XGBoost

XGBoost is a widely used implementation of gradient boosting that is both scalable and efficient. It incorporates several techniques that optimize the training process, such as parallel processing, approximate tree learning, and regularized boosting^33^.

##### LightGBM

LightGBM is a highly optimized implementation of gradient boosting that is specifically designed for handling large-scale datasets. It shares some similarities with XGBoost in terms of its approach but incorporates additional optimizations such as histogram-based gradient boosting and leaf-wise tree growth^33^.

##### CatBoost

CatBoost is a boosting algorithm that was developed relatively recently, and it is specifically designed to handle categorical variables in a more effective way than other boosting algorithms. It achieves this by converting categorical variables into numerical features using various encoding techniques, and then using gradient boosting to train a model on these features^34^.

#### Stacking

Stacking is a machine learning technique that involves training multiple models and using their predictions as input features for a final model. The final model is then trained on these predictions to obtain the final prediction. Ensemble learning, which includes techniques like stacking, has been shown to be highly effective in improving the accuracy and robustness of machine learning models, and it has been successfully applied to a wide range of applications^34^.

##### XGBOOST

**Figure 3:**
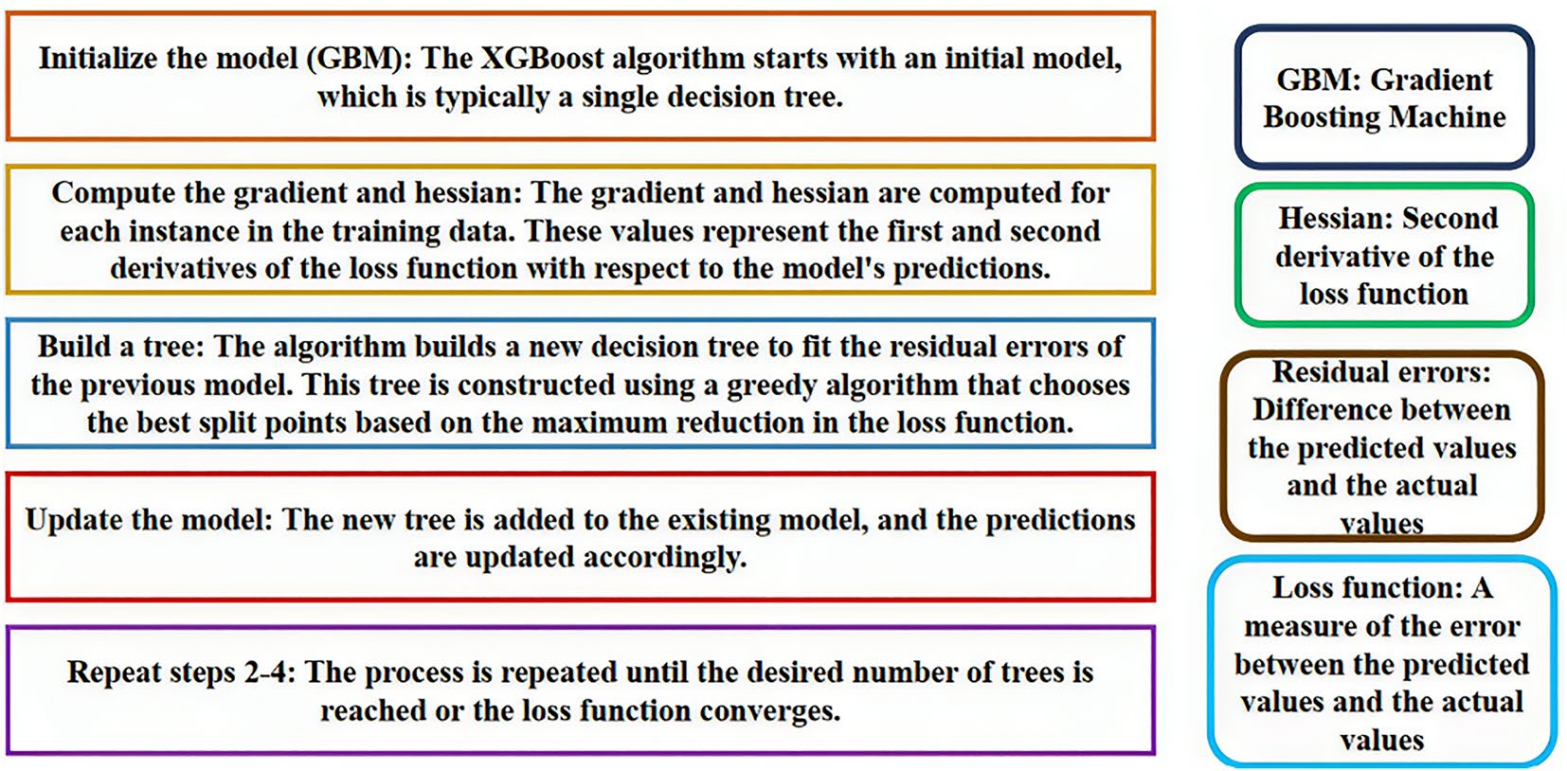
Abbreviation-based explanation of the XGBoost algorithm^35^.

**Figure 4:**
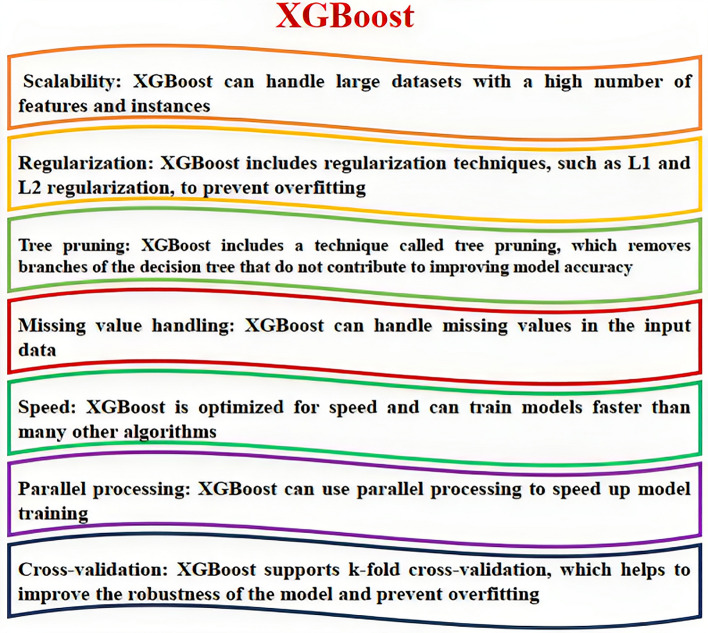
Key features of the XGBoost method.

XGBoost is a powerful and popular ensemble learning method in the field of machine learning. It is a type of boosting method that combines several weak learners to create a strong learner. The basic idea behind XGBoost is to iteratively add new models to the ensemble, with each new model attempting to correct the errors made by the previous models^35^. The XGBoost algorithm is based on decision trees, which are simple models that can be combined to form more complex models. In XGBoost, each tree is trained to predict the residual errors of the previous trees, rather than the actual target values. This approach helps to reduce the bias of the ensemble and improve its predictive power. One of the key features of XGBoost is its ability to handle missing data and outliers. It uses a technique called gradient boosting to update the model parameters, which allows it to handle missing data by assigning weights to the missing values. It also uses regularization techniques to prevent overfitting and improve the generalization performance of the model^35^. A schematic and abbreviation-based explanation of the XGBoost algorithm is shown in Fig. 3, also you can see some key features of XGBoost in Fig. 4.

In the field of engineering, XGBoost is widely used. Some of its applications are described briefly.

In study by Fang et al.^36^ XGBoost was employed to develop a machine learning model for assessing the web crippling behavior of perforated roll-formed aluminum alloy (RFA) unlipped channels. This model, trained on data points generated from finite element analysis, exhibits a remarkable prediction accuracy, surpassing other methods like Random Forest and Linear Regression.

Another study by Dai et al.^37^ used XGBoost to evaluate the moment capacity of cold-formed steel (CFS) channel beams with edge-stiffened web holes subjected to bending. This novel machine learning model is trained on data points, generated using an elastoplastic finite element model, validated against existing literature data. In addition, XGBoost was applied by Lim and Chi^38^ in bridge management to estimate the condition of bridges and predict potential damage. This method is chosen for its ability to handle a multitude of variables without making strong assumptions about determinacy and independence, making it a suitable tool for this complex task. In addition, XGBoost has shown accurate predictions for some cases in the field of petroleum engineering, such as the prediction of oil formation volume factor and viscosity^39,40^.

### Input parameters and data

To assess the conductivity of etched fractures under conditions that are thought to be representative of field conditions, Nierode and Kruk^13^ developed a testing procedure. The test involves breaking a core plug in tension to simulate the rough surface found in the field, acidizing it as a vertical fracture, subjecting it to a closure stress, and measuring the conductivity. The findings of these tests are used in this study. Additionally, the findings of these tests and a representative plot of conductivity versus closure stress for a San Angelo Dolomite core plug are available in Nierode and Kruk^13^.

So, a total of 116 experimental data points were collected to develop a more accurate XGBoost model for predicting fracture conductivity. The input features for each sample were DREC, RES, and closure stress, while fracture conductivity was used as the target variable, means that there are three columns as an input and one column as an output for the dataset. The paper utilized accurate methods for determining outliers, noise, and model robustness.

To obtain more information about the dataset refer to Nierode and Kruk^13^.

## Result and discussion

### Outlier box plot

**Figure 5:**
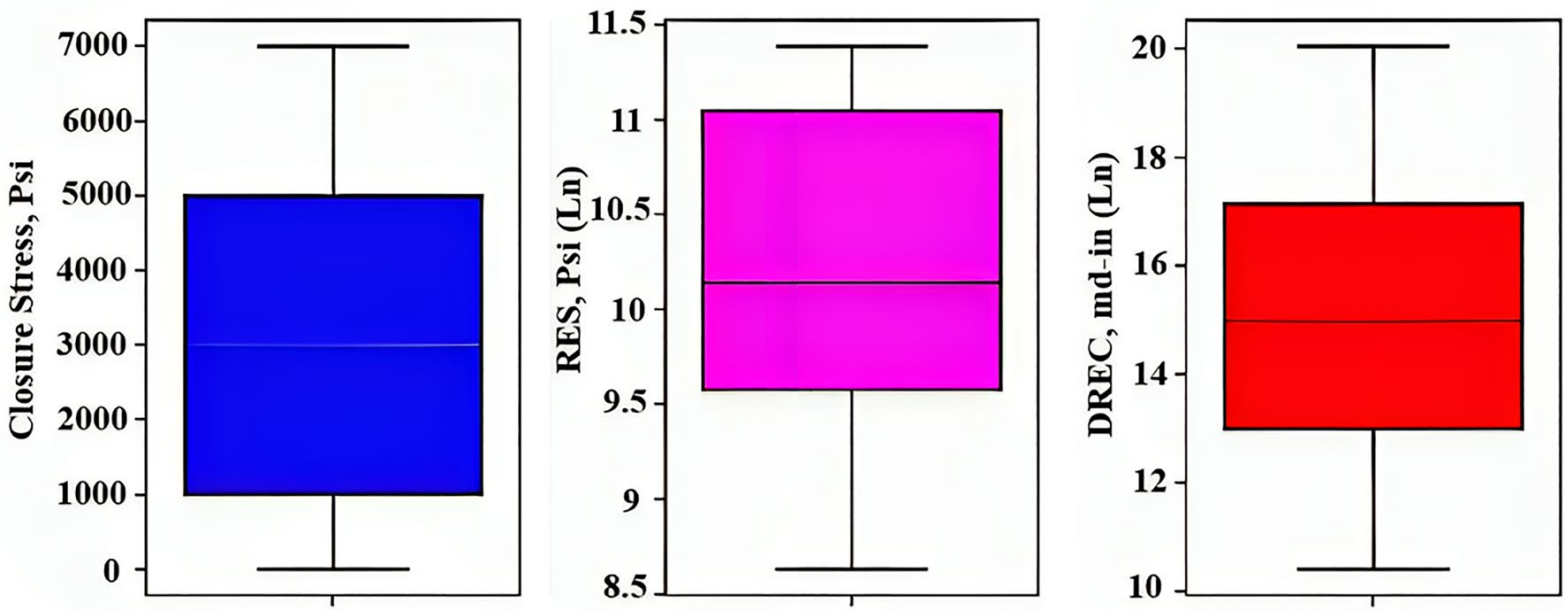
Visual representation of the quartiles and the range of the data.

Figure 5 provides a visual representation of the quartiles and range of the data. The box in the box plot represents the interquartile range (IQR), which is the difference between the 75th and 25th percentiles of the data. The line inside the box represents the median. The whiskers extend to the highest and lowest data points that are within 1.5 times the IQR from the upper and lower quartiles, respectively.

If any data points fall outside the whiskers, they are plotted as individual points and are considered outliers. These outliers can be easily identified on the box plot by looking for individual points outside the whiskers. Figure 5 can also help identify the position of the outliers relative to the rest of the data. If there are outliers on the upper end of the box plot, it indicates that the data has a long tail on the right-hand side and is positively skewed. On the other hand, if there are outliers on the lower end of the box plot, it indicates that the data has a long tail on the left-hand side and is negatively skewed. According to this figure, there are no outliers for this dataset because there are no data points outside the box plots.

### Violin plot

**Figure 6:**
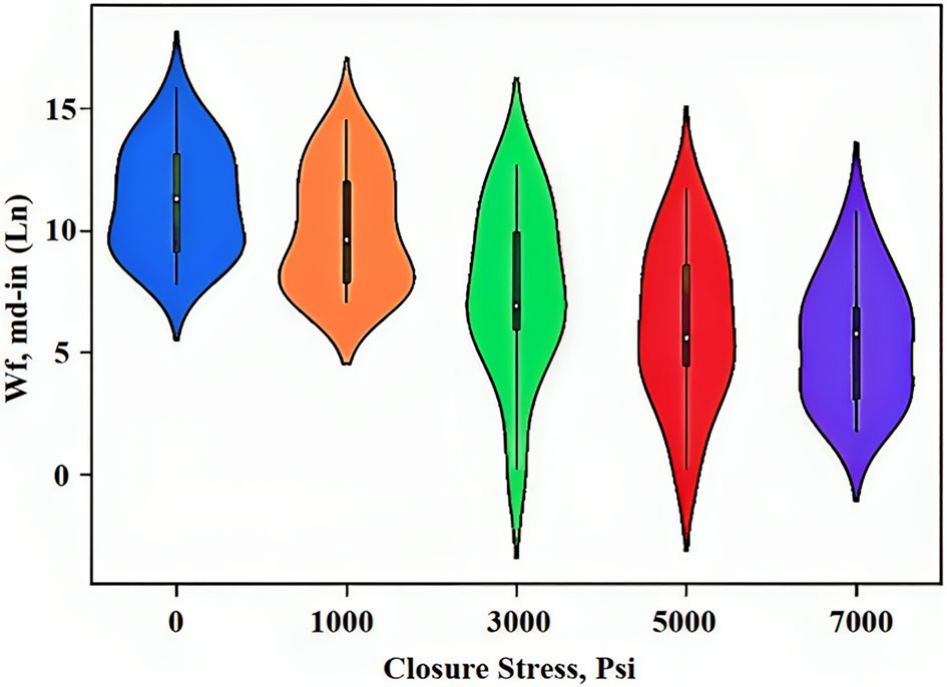
Distribution of numerical data in different closure stresses*.*

The purpose of Fig. 6 is to show the distribution of numerical data at different closure stresses. It's similar to a box plot in that it displays the median, quartiles, and outliers of a dataset, but it also includes information about the density of the data at different values. The shape of the "violin" in the plot is created using a statistical technique called kernel density estimation, which estimates the probability density function of a variable. The width of the violin at a given point represents the density of the data at that point.

### Pair plot

**Figure 7:**
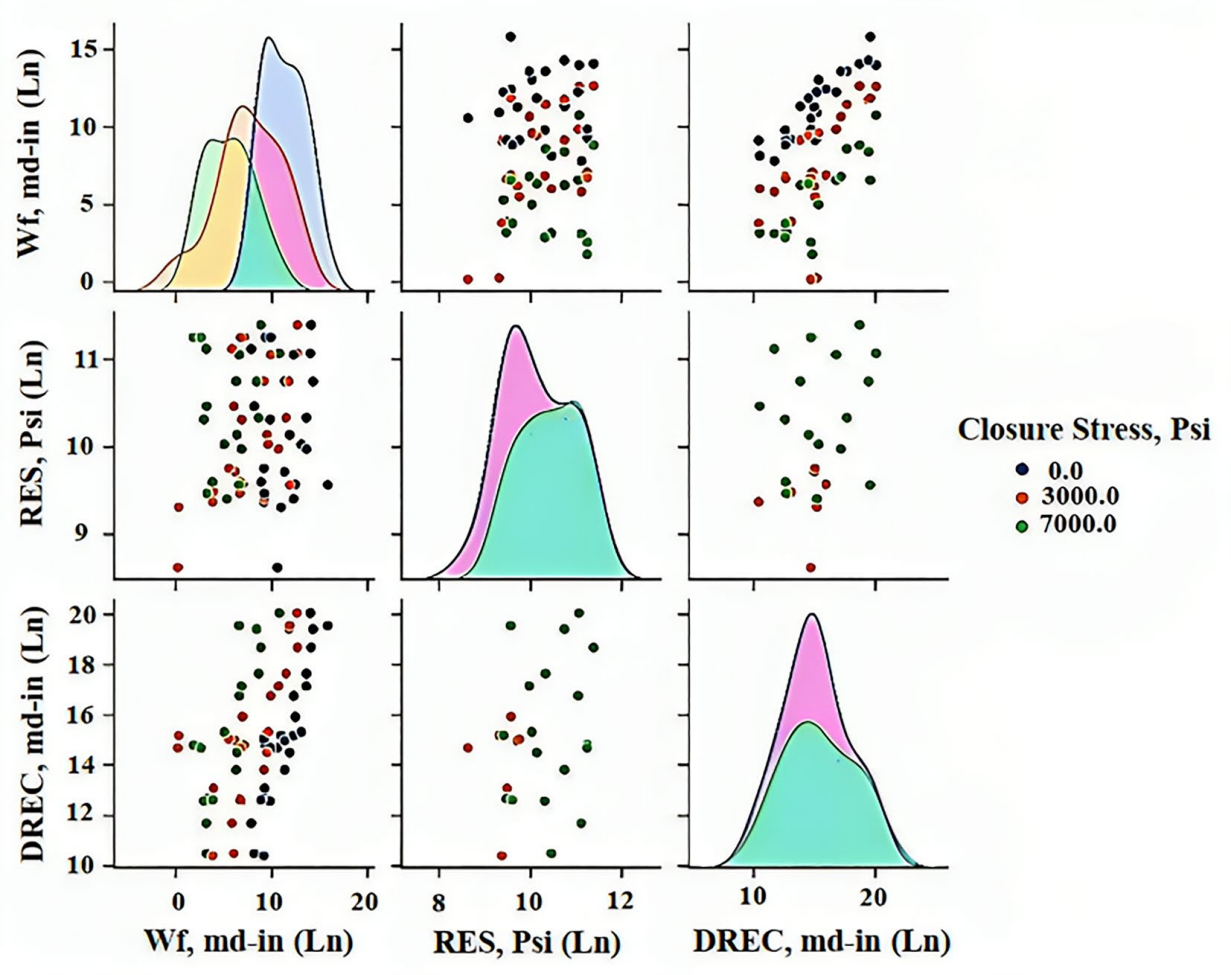
Pair plot showing scatter plots and histograms of the dataset variables.

Figure 7 displays a grid of scatter plots and histograms, where each variable is plotted against every other variable in a dataset consisting of four variables (one of them being the target variable). By excluding the closure stress variable and using it to separate the dataset into three different colors based on their closure stress values, the figure would show nine plots, with scatter plots occupying the upper right diagonal and histograms occupying the lower left diagonal. The scatter plots in the pair plot provide information about the relationship between two variables, such as whether they are positively correlated (i.e., they both increase or decrease together), negatively correlated (i.e., one variable increases while the other decreases), or uncorrelated (i.e., there is no clear pattern). The histograms in the pair plot show the distribution of each variable, indicating whether it is normally distributed (i.e., follows a bell curve), skewed to the left or right (i.e., has a tail that goes off in one direction), or has multiple peaks (i.e., is bimodal or multimodal). This figure allows us to explore the relationships between multiple variables in a dataset and identify patterns and trends that may warrant further investigation.

### Heat map

**Figure 8:**
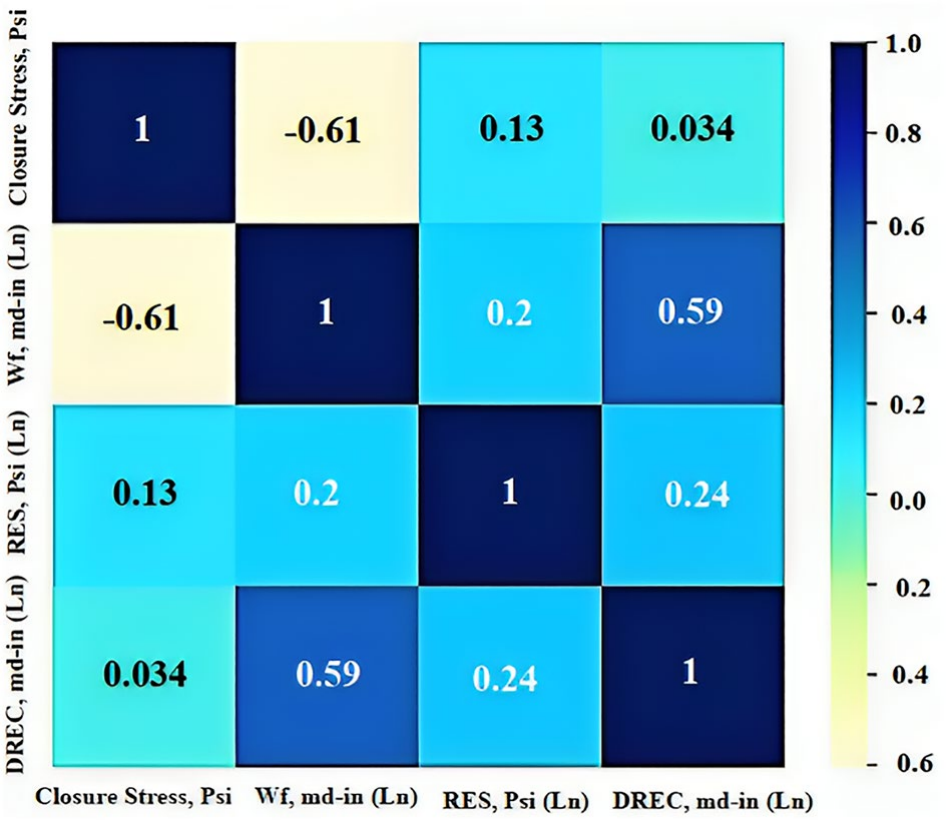
Pearson correlation.

Figure 8 illustrates the Pearson correlation, which is a statistical method utilized to measure the direction and strength of the linear relationship between two variables. The correlation result is a value ranging from − 1 to 1, where a value of 1 indicates a perfect positive correlation, a value of − 1 indicates a perfect negative correlation and a value of 0 indicates no correlation between the variables. It's essential to note that correlations greater than (0.3–0.4) or less than ((− 0.3) to (− 0.4)) are generally not accepted since inputs should be independent. Based on the heat map, all of the inputs or features are independent from each other. This is because none of them have a correlation value greater than 0.4 or less than − 0.4.

### Parameters and hyperparameters

Tables 1, 2, and 3 provide an explanation of various statistical measures, including count, mean, standard deviation, minimum, maximum, and quartiles. These measures are important for understanding the characteristics of the data set and identifying patterns or trends.

**Table 1:** Various statistical measures for understanding the characteristics of the data set and identifying patterns or trends for all dataset.

Parameters	Closure stress (psi)	W_f_, md-in (Ln)	DREC, md-in (Ln)	RES, psi (Ln)
Count	116	116	116	116
Mean	2939.65	8.33	15.13	10.21
Std	2475.22	3.50	2.67	0.72
Min	0	0.18	10.40	8.63
25%	1000	6.23	12.98	9.57
50%	3000	8.58	14.97	10.13
75%	5000	10.95	17.14	11.04
Max	7000	15.81	20.04	11.38

**Table 2:** Various statistical measures for understanding the characteristics of the data set and identifying patterns or trends for limestone.

Parameters	Closure stress, psi	W_f_, md-in (Ln)	DREC, md-in (Ln)	RES, psi (Ln)
Count	55	55	55	55
Mean	3200	9.18	15.62	10.24
Std	2584.85	3.30	2.87	0.60
Min	0	2.89	11.69	9.47
25%	1000	6.74	12.61	9.60
50%	3000	8.85	15.31	10.13
75%	5000	11.84	18.68	10.74
Max	7000	15.81	19.55	11.38

**Table 3:** Various statistical measures for understanding the characteristics of the data set and identifying patterns or trends for dolomite.

Parameters	Closure stress, psi	W_f_, md-in (Ln)	DREC, md-in (Ln)	RES, psi (Ln)
Count	51	51	51	51
Mean	2901.96	7.94	14.79	10.38
Std	2443.39	3.29	2.60	0.74
Min	0	0.26	10.40	9.37
25%	1000	5.80	13.81	9.64
50%	3000	8.13	14.97	10.46
75%	5000	10.59	15.93	11.06
Max	7000	13.99	20.04	11.24

The "count" measure indicates the total number of observations in each column, which helps to understand the completeness and size of the data set.

The "mean" measure represents the average value of each column, calculated by dividing the sum of all values in the column by the total number of observations. The mean provides insight into the central tendency of the data. It is worth noting that the experimental data were randomly split into two groups: an 80% training group and a 20% testing group. This approach has been proven to be effective and reliable for producing accurate predictions for unknown cases.

To prevent overfitting in XGBoost, tuning hyperparameters is essential. Parameters such as the learning rate, tree depth, and the number of boosting rounds can be adjusted to strike a balance between model complexity and generalization. A lower learning rate, for instance, can slow down the learning process, enabling the model to generalize better. Similarly, controlling tree depth and the number of boosting rounds can help avoid capturing noise in the training data^33^.

In this study, three XGBoost algorithms were utilized for all data, as well as for dolomite and limestone separately. Therefore, three XGBoost methods with different hyperparameters were built to train these models, and Table 4 displays the hyperparameters for each XGBoost algorithm.

**Table 4:** Hyperparameters for each XGBoost algorithm.

Models	N estimators	Learning rate	Sub sample	Col-sample by tree	Max depth
XGBoost (all data)	80	0.075	0.75	1	15
XGBoost (dolomite)	100	0.07	0.75	1	7
XGBoost (limestone)	70	0.09	0.75	1	9

Dolomite and limestone are selected for analysis and comparison to study the effect of lithology on fracture conductivity. One of the models considered only dolomite data, and another model used only limestone rock type.

The XGBoost model has several parameters that are defined as follows:

- n-estimators: this parameter determines the number of decision trees that will be created during the boosting process. Increasing the value of n-estimators will generally improve the model's performance, but it will also increase the training time.
- Learning-rate: this parameter controls the weight assigned to each tree in the ensemble. A lower learning rate will make the model more resistant to overfitting, but it will also increase the training time.
- subsample: this parameter determines the fraction of observations that are randomly sampled for each tree. A value of 1.0 indicates that all observations are used for each tree, while a smaller value will result in faster training times, but may also lead to underfitting.
- Col sample-by tree: this parameter controls the fraction of features that are randomly sampled for each tree. A smaller value will lead to faster training times, but may also result in underfitting.
- Max-depth: this parameter determines the maximum depth of each decision tree in the ensemble. A deeper tree will be able to capture more complex relationships in the data, but it will also be more susceptible to overfitting^41^.

To measure the model performance some regression metrics are defined as follows:

1. Average absolute relative error (AARE).
  1 $$AARE\%=\frac{1}{N}\sum_{i=1}^{N}\left|\frac{{W}_{f(iexp)}-{W}_{f(ipred)}}{{W}_{f(iexp)}}\right|\times 100$$
  AARE stands for average absolute relative error, which is a statistical measure used to evaluate the accuracy of a forecasting model. It is calculated by taking the absolute value of the difference between the actual and predicted values, dividing the result by the actual value, and then taking the average of these values. A lower AARE value indicates a more accurate forecasting model.
2. Coefficient of determination (*R*^*2*^).
  2 $$ {\text{R}}^{2}  = 1 - \frac{{\sum _{{i = 1}}^{N} \left( {W_{{f(iexp)}}  - W_{{f(ipred)}} } \right)^{2} }}{{\sum _{{i = 1}}^{N} \left( {W_{{f(ipred)}}  - \overline{{W_{f} }} } \right)^{2} }} $$
  The coefficient of determination (*R*^*2*^) is a statistical measure used to evaluate the goodness of fit of a regression model. It is calculated by squaring the correlation coefficient (r) between the actual and predicted values. The resulting value (*R*^*2*^) ranges from 0 to 1, where 0 indicates no correlation and 1 indicates perfect correlation. A higher *R*^*2*^ value indicates a better fit of the regression model.
3. Root mean square error (RMSE).
  3 $$RMSE=\sqrt{\frac{1}{N}\sum_{i=0}^{N-1}{{(W}_{f(iexp)}-{W}_{f\left(ipred\right)})}^{2}}$$
  Root mean square error (RMSE) is a statistical measure used to evaluate the accuracy of a forecasting model. It is calculated by taking the square root of the mean of the squared differences between the actual and predicted values. A lower RMSE value indicates a more accurate forecasting model.

Equations (1–3) use ${{\text{W}}}_{{\text{f}}}$ to indicate the output, which is related to fracture conductivity. exp and pred refer to the actual and estimated values of fracture conductivity, respectively. Moreover, $\overline{{{\text{W}} }_{{\text{f}}}}$ represents the average of the outputs, and N represents the number of data points.

In addition, by using analysis, conducted graphical evaluations to visually demonstrate how well the models were able to predict fracture conductivity were conducted with accuracy and efficiency.

### XGBoost algorithm

#### For all dataset

**Figure 9:**
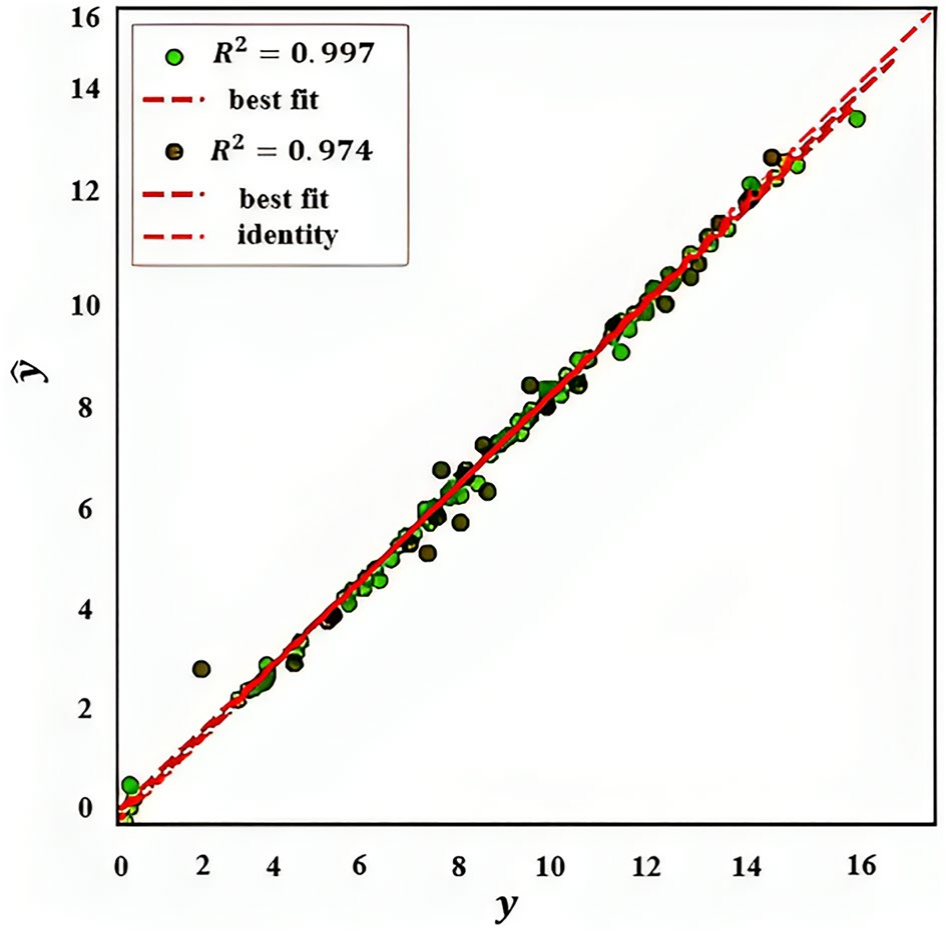
Evaluating the performance of a regression model (prediction error for XGB regressor).

In Fig. 9, a regression model's performance is evaluated by plotting the predicted values on the y-axis and the actual values of the target variable on the x-axis. The purpose of this plot is to assess the accuracy of the model's predictions. The ideal scenario is that the predicted values fall exactly on the diagonal line, which represents the identity line, indicating that the predicted values are identical to the actual values. However, in reality, most models are not perfect, and some deviation between the predicted and actual values is expected.

The plot includes a line of best fit, which shows the overall trend in the data. The closer the predicted values are to this line, the better the model is performing. The identity line displays a diagonal line where the predicted values are equal to the actual values, which is useful for evaluating the accuracy of the model's predictions. A residual plot is a useful tool that displays the difference between predicted and actual values (residuals) on the y-axis and the actual values on the x-axis. y: actual data points, $\widehat{y}$: predicted data points.

To show the model’s prediction deviation better, the residual figure is used as follows.

**Figure 10:**
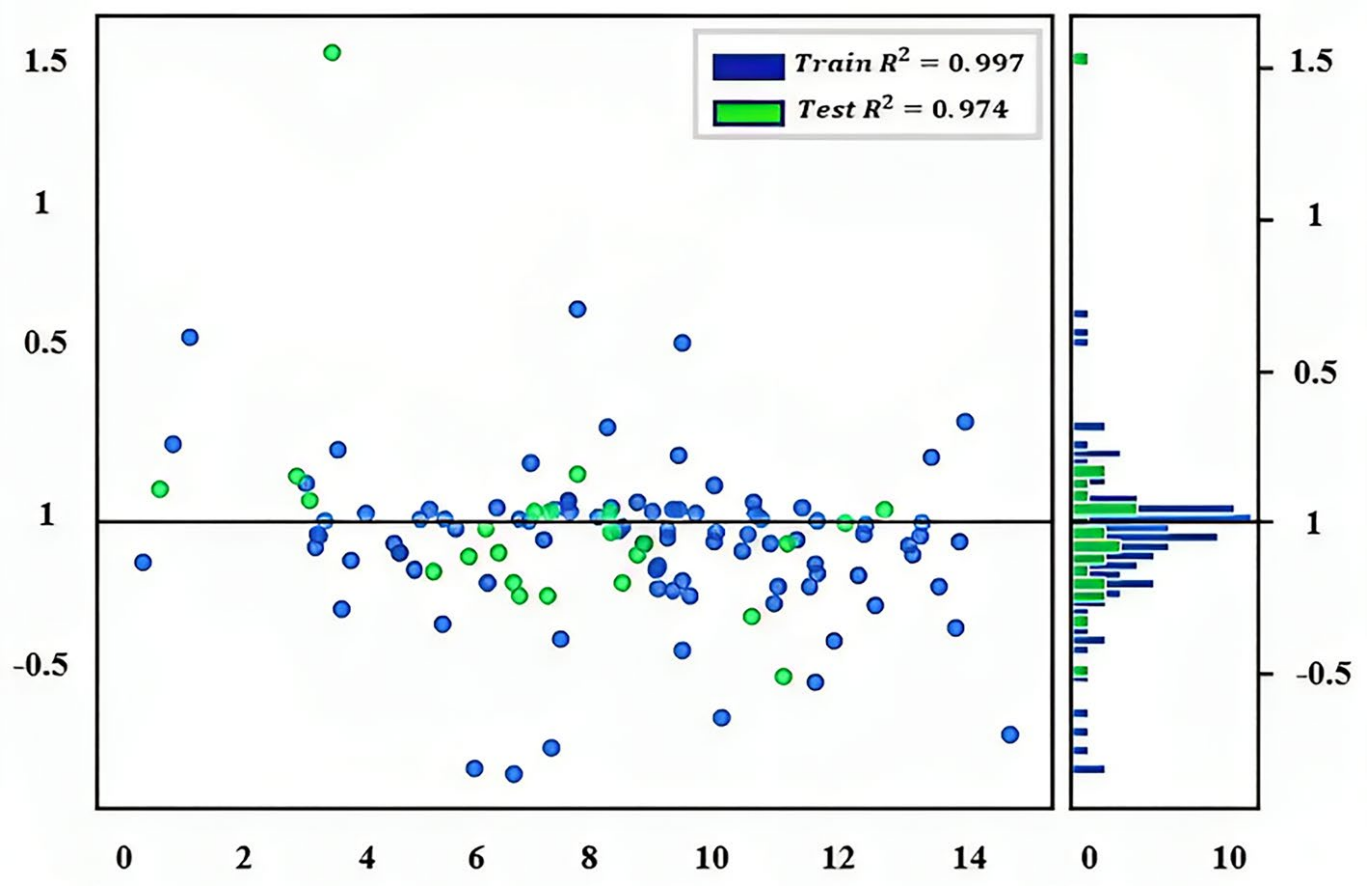
Residuals for XGB regressor model.

Figure 10, known as the residual plot, displays the difference between the predicted values and the actual values of a model, which are also known as the "residuals" or errors of the model. This figure is useful for identifying patterns in the residuals, which can aid in diagnosing issues with the model, such as heteroscedasticity or nonlinearity. The residuals are plotted on the y-axis, and the predicted values are on the x-axis. Additionally, a horizontal line is included at y = 0, representing the perfect prediction line. If the residuals are randomly scattered around the y = 0 line, then the model is making accurate predictions. It is important to note that this figure does not show the feature importance or which input has the most effect on the model's prediction. It only displays the residuals of the model. Feature importance is typically displayed in other types of plots, such as a bar graph or heat map.

**Figure 11:**
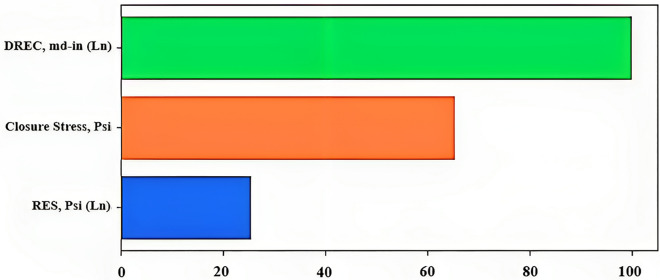
Horizontal bar chart showing the importance scores of each feature in the dataset.

Figure 11 is a horizontal bar chart that displays the importance scores of each feature in a dataset. The height of each bar represents the importance score of the corresponding feature, with the most important feature at the top of the plot. The features are arranged in descending order of importance.

The importance scores are calculated using a permutation-based method, where each feature is randomly shuffled, and the decrease in model performance is measured. The difference in performance before and after shuffling the feature is used as the feature importance score.

Interpreting the plot involves identifying the most important features based on their height and potentially selecting a subset of the most important features for use in subsequent modeling or analysis. This plot provides valuable insights into the inner workings of the XGBoost model and helps to understand which features are driving model performance.

#### For dolomite and limestone

**Figure 12:**
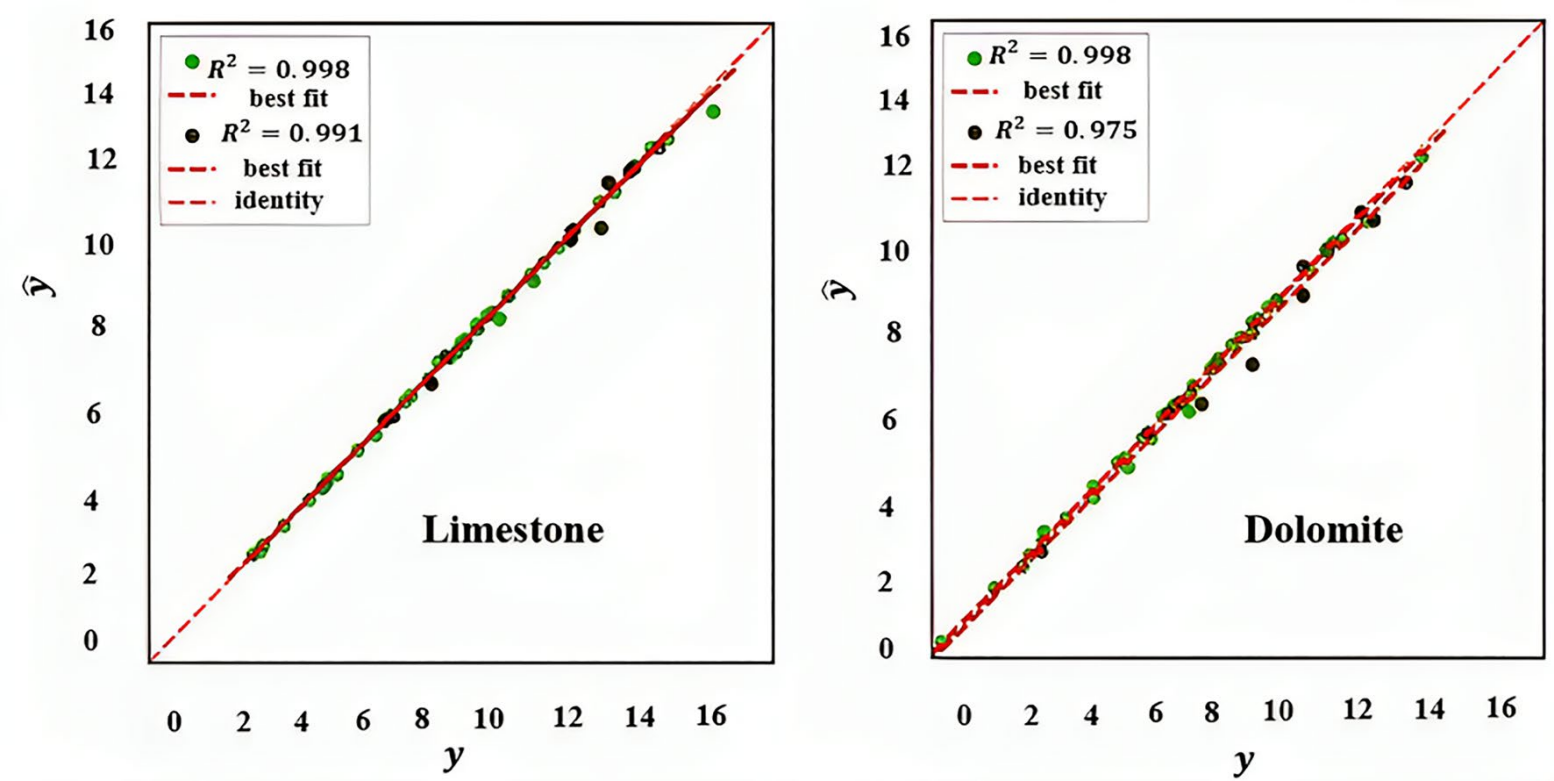
Evaluating the performance of a regression model for limestone and dolomite (prediction error for XGB regressor).

Evaluating the performance of a regression model for dolomite and limestone is shown in Fig. 12. By analyzing R^2^-score for test data, it can be concluded that XGBoost for limestone is stronger than dolomite. This suggests that the model can predict fracture conductivity for limestone more accurate than dolotime. The reason is that the model is better at fitting on limestone rocks dataset than fitting on dolomite rocks dataset.

### Sensitivity analysis

#### Sensitivity analysis based on DREC.

**Figure 13:**
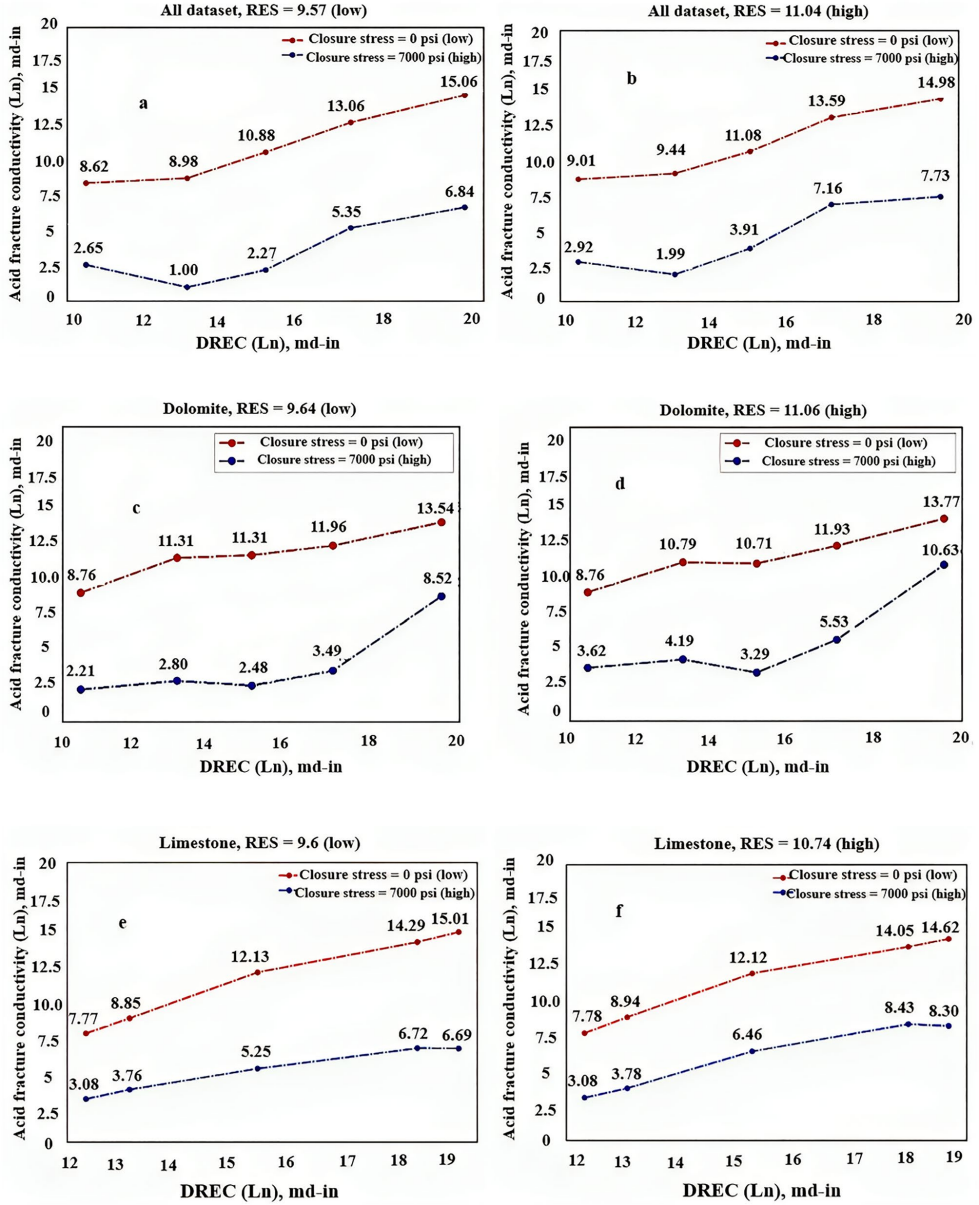
Sensitivity analysis of acid fracture conductivity* for different rock types based on DREC.*

The sensitivity analysis of acid fracture conductivity for different rock types (all dataset (a), dolomite (b), limestone (c)) was performed by varying the DREC values under different closure stress levels and RES values (low and high) Fig. 13.

#### Sensitivity analysis based on closure stress

**Figure 14:**
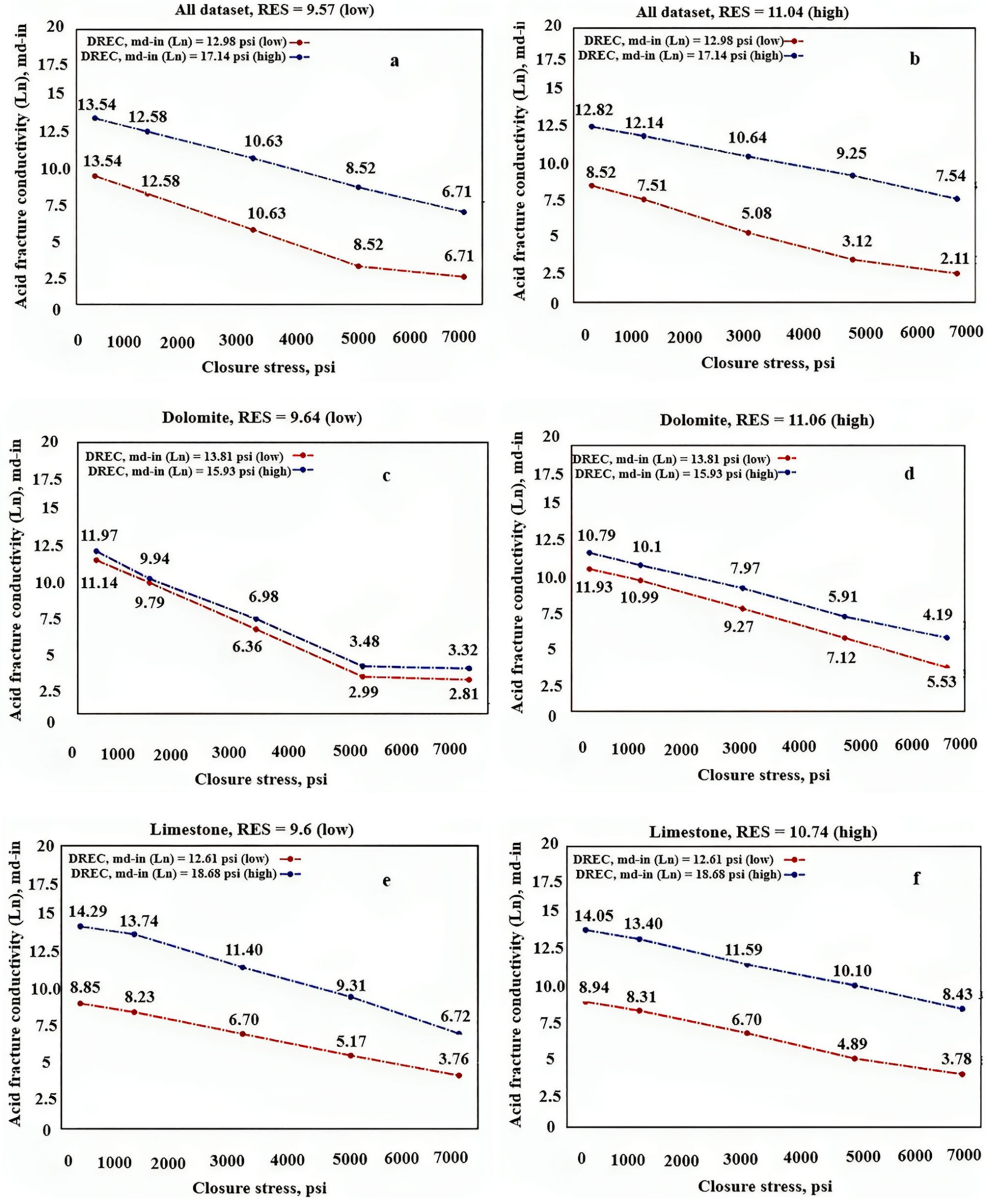
Sensitivity analysis of acid fracture conductivity for different rock types based on closure stress.

Sensitivity analysis of acid fracture conductivity for different rock types (all dataset (a), dolomite (b), limestone (c)) based on closure stress for different DREC and RES values (low and high) is being discussed Fig. 14.

By analyzing the sensitivity analysis performed in both sections, the following results can be obtained:

1. It is anticipated that an increase in closure stress will result in a decrease in conductivity, a trend that was observed across all graphs.
2. With regards to DREC, a parameter that indicates the strength, concentration, and volume of acid used, an increase in operational costs is expected. However, if this increase leads to a significant rise in conductivity, it is deemed worthwhile. As shown in Fig. 13, an increase in DREC is expected to increase conductivity, a trend that was observed in almost all graphs.
3. RES is a mechanical parameter of rock that represents its strength and resistance. Fractures created by acid reactions on rock surfaces must have sufficient strength against closure stress, which depends on the rock's RES. The higher the RES, the stronger the rock and the more resistant it is to flow. As RES increases, conductivity also increases, resulting in a combined effect observed in most graphs.
4. Conductivity decreases as closure stress increases, and the type of decrease indicates the model's strength. Exponential trends were found in examining these changes, which are related to the behavior of the rock.
5. As per the acid fracturing process theory, an increase in DREC leads to a wider dissolution of rocks, which creates more pathways and ethes for fluid flow. This results in an increase in fracture conductivity. However, the trend of conductivity increase varies across different rock types, indicating the influence of rock type on the developed models. It is not advisable to present a comprehensive model to predict conductivity behavior as it may result in irrational model behavior. Therefore, it is recommended to divide the developed models based on rock types.
6. The conductivity value in dolomites and limestones is expected to increase with an increase in DREC. The increase in conductivity in dolomites is higher at higher closure stresses compared to lower closure stresses. Conversely, this behavior is reversed in limestones. At zero closure stress, the increase in conductivity for limestones per unit increase in DREC is greater than that for dolomites, indicating a higher reaction rate of acid with limestone samples compared to dolomite samples. However, at a closure stress of around 7000 psi, the increase in conductivity for dolomites per unit increase in DREC is greater than that for limestones, indicating the high strength of dolomite samples compared to limestone samples under high closure pressures. Therefore, it is recommended to increase conductivity with an increase in DREC in limestone formations with low closure stress, while in dolomite formations at high closure stress, an increase in DREC may not result in a significant increase in conductivity, except at very high closure stresses that require pre-testing of fracture conductivity before operations.
7. The strength of rock has a significant impact on conductivity under high closure stresses. As rock strength increases, the formation's ability to resist crushing increases, maintaining pathways for fluid flow even under high closure stress. This results in higher conductivity compared to samples with lower rock strength under similar closure stresses.
8. Acid fracturing theory confirms that pathways created by the acid-rock reaction gradually close as closure stress increases, reducing the fracture's ability to allow fluid flow. As a general trend, fracture conductivity decreases. However, the conductivity decrease in different rock types may not follow the same trend. This emphasizes the importance of considering rock type in modeling. Developing a unified model to predict conductivity behavior may result in errors and irrational model behavior (Fig. 14a). Therefore, it is suggested to develop separate models based on rock types and avoid presenting a comprehensive model for different rock types.
9. The analysis of images Fig. 14c–f suggest that the impact of increased DREC on conductivity is greater in limestone samples than in dolomite samples.
10. When rock strength (RES) is higher, fissures tend to close more, resulting in greater fluid permeability through fractures compared to samples with lower RES (see Fig. 14c–f).
11. Based on the presented models by rock type, there was a higher decrease in conductivity due to an increase in closure stress observed in dolomite samples compared to limestone samples. For example, with an increase in specific closure stress value, dolomite samples showed an approximately 70% reduction in conductivity, while limestone samples exhibited around a 50% reduction. However, in dolomite samples with low RES, when closure stress exceeded 5000 psi, the change in conductivity showed no significant variation with increasing closure stress (see Fig. 14c). This phenomenon could be attributed to the crushing effect that occurs due to the low strength of dolomite samples at low RES. In dolomite samples with high strength (high RES), a decreasing trend in conductivity was observed with increasing closure stress (see Fig. 14d).
12. In low-strength dolomite samples, increasing DREC does not significantly enhance conductivity (see Fig. 14c), and it only adds to the operational expenses of fracturing projects. However, beyond a specific threshold value (known as critical DREC), an increase in conductivity can be substantial (see Fig. 13c). Determining this critical DREC is challenging but feasible through laboratory experiments to measure fracture conductivity. For the dolomite samples used, the critical DREC value is set at 17. However, this phenomenon was not observed in high-strength dolomite samples (see Fig. 14d), where conductivity increases with an increase in DREC. Nevertheless, this increase is much less significant compared to limestone samples, indicating the significant effect of DREC on conductivity in limestone samples (see Fig. 14e,f). Therefore, it is recommended to increase DREC in fracturing processes for limestone formations as much as operational conditions allow. However, for dolomite formations, taking into account rock strength, it is crucial to determine the critical DREC value before initiating the process and then proceed with increasing DREC accordingly.
13. The XGBoost Dolomite model predicts a nonlinear increase in conductivity under various DREC, with the rate of increase slightly increasing as closure stress increases. Dolomite conductivity is observed to be more sensitive to treatment parameters at high closure stress than at low closure stress. The XGBoost Limestone model predicts a similar nonlinear behavior of rock versus DREC under various closure stresses.

#### Comparison

Table 5 suggests that the XGBoost model has a better performance on specific rock types compared to the combined rock data. The model trained on limestone data generally has lower errors compared to the model trained on all data. On the other hand, the errors associated with the model trained on dolomite data are generally higher than those associated with the model trained on limestone data. This finding supports the idea that the XGBoost model may be more suitable for predicting the properties of limestone rocks compared to dolomite rocks.

**Table 5:** Comparison of the result of this approach for different rock types.

Models	Train RMSE	Train R^2^	Train AARE (%)	Test RMSE	Test R^2^	Test AARE (%)	Overall RMSE	Overall R^2^	Overall AARE (%)
XGBoost (all data)	0.1800	0.9973	6.0046	0.5187	0.9744	7.6674	0.2477	0.9927	6.3371
XGBoost (dolomite)	0.1619	0.9975	3.8178	0.4839	0.9747	3.9980	0.2263	0.9929	3.8538
XGBoost (limestone)	0.1402	0.9981	0.8562	0.2630	0.9908	1.5229	0.1647	0.9966	0.9895

Therefore, it is concluded that the XGBoost model performs better on specific rock types compared to the all data. This may be due to the fact that the XGBoost model is able to capture the unique characteristics and patterns of specific rock types, leading to more accurate predictions.

### Comparison of this approach with previous correlations and intelligent methods

The results of this study have been compared to the results of the studies conducted by the individuals mentioned in Table 6.

**Table 6:** Comparison of the result of this approach with previous correlations and studies.

Year models	Train RMSE	Train R^2^	Train AARE (%)	Test RMSE	Test R^2^	Test AARE (%)	Overall RMSE	Overall R^2^	Overall AARE (%)
This study XGBoost	0.1800	0.9973	6.0046	0.5187	0.9744	7.6674	0.2477	0.9927	6.3371
Yaser Motamedi et al.^23^	NAN	NAN	NAN	NAN	NAN	NAN	NAN	0.85	34.55
Akbari Mohammadreza, et al.^25^	NAN	NAN	NAN	NAN	NAN	NAN	NAN	0.85	34.55
Akbari Mohammadreza, et al.^24^	NAN	0.9969	NAN	NAN	0.9446	8.54	NAN	0.9852	NAN
N&K^13^	NAN	NAN	NAN	NAN	NAN	NAN	NAN	0.77	60.52
NSD^15^	NAN	NAN	NAN	NAN	NAN	NAN	NAN	0.81	53.55

Based on the information provided in the table above, the XGBoost model outperforms the other models in terms of both training and testing metrics. The XGBoost model has the lowest overall RMSE of 0.2477, which indicates that it has the highest accuracy in predicting the conductivity of rocks. Additionally, the XGBoost model has the highest overall R^2^ (coefficient of determination) of 0.9927, which indicates a strong correlation between the predicted and actual conductivity values. Furthermore, the XGBoost model has the lowest overall AARE of 6.3371%, which indicates that it has the lowest average percentage error compared to the other models. Therefore, the XGBoost model is considered to be superior to the other models in predicting rock conductivity.

One reason why XGBoost may have performed better than ANN in Akbari et al.^24^ is that machine learning methods generally perform better than deep learning methods when the dataset is small. This is because deep learning models tend to have many parameters and require a large amount of data to learn meaningful patterns and avoid overfitting.

**Figure 15:**
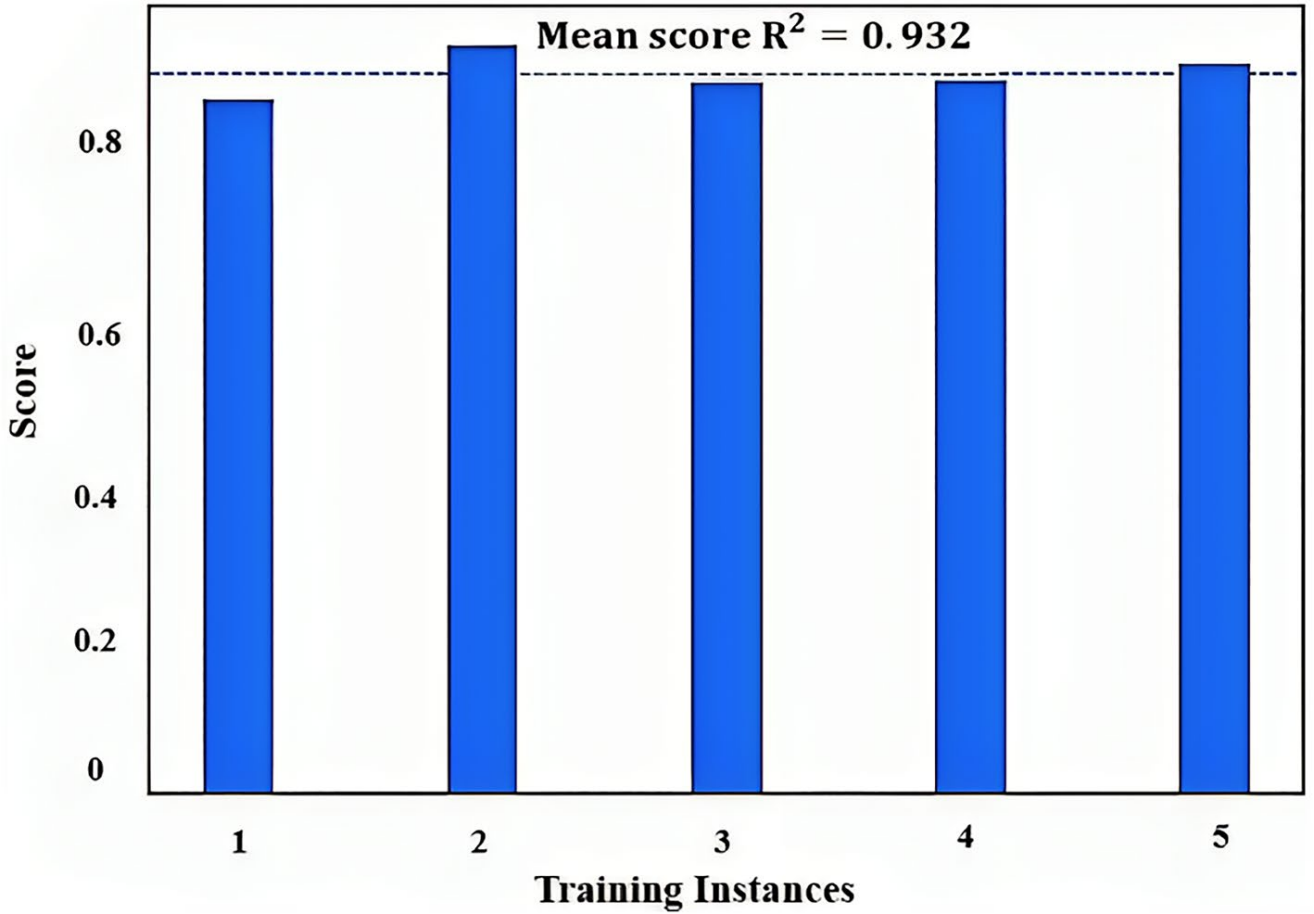
Cross-validation scores for XGB regressor.

Previous studies have ignored the model's performance for other data points used for training, which can create uncertainties for the algorithms used. To demonstrate XGBoost's robustness, k-fold cross-validation was used, where k is the number of folds. In 5-fold cross-validation Fig. 15, the dataset is randomly divided into 5 equal parts, and the model is trained and evaluated 5 times, with each of the 5 parts used once as the validation data and the other 4 parts used as the training data.

A 0.932 accuracy score was achieved in fivefold cross-validation, indicating that the model correctly predicted the target variable for 93.2% of the data points in the validation set. However, it's important to keep in mind that cross-validation provides an estimate of the model's performance on unseen data, and the actual performance of the model may differ when applied to new data.

By comparing the cross-validation accuracy score and value for the test dataset, it can be inferred that XGBoost does not suffer from overfitting, as there is a low difference between them.

## Conclusion

### Importance of fracture conductivity prediction

The study underscores the crucial role of XGBoost in intelligently modeling acid fracture conductivity predictions, offering a platform to optimize acid fracturing treatments. Understanding the diverse impact of rock type and treatment parameters on fracture conductivity is pivotal for enhancing well productivity. The results present a potential means to maximize fracture conductivity through tailored acid fracturing treatments, pointing the way to more efficient resource extraction.

### Advantages of XGBoost and comparison with other methods

The robustness and efficiency of XGBoost are emphasized, particularly in its ability to handle high-dimensional data and missing values. The comparison with the ANN Akbari model unveils XGBoost's superior performance in reducing errors and increasing the predictive power for acid fracture conductivity. Additionally, the model's ability to avoid overfitting is a key advantage, attributed to early stopping, the learning rate parameter, and data subsampling features, thereby contributing to its reliability and accuracy.

### Sensitivity analysis

The sensitivity analysis offers profound insights into the impact of various parameters on fracture conductivity, elucidating the complex behavior of rock formations under diverse conditions. By considering closure stress, DREC, and RES, the study highlights the necessity of integrating these parameters into the design of acid fracturing operations, with substantial implications for scheduling and fluid selection, ultimately optimizing the prediction and design of acid fracturing treatments.

## Rock types effect

The study emphasizes the criticality of tailoring modeling and analysis based on rock types to optimize acid fracturing operations and enhance understanding of fracture behavior in diverse formations. Insights into parameters such as closure stress, DREC, and rock strength underscore the need for meticulous pre-testing and the consideration of specific formation characteristics to ensure accurate predictions before operational implementation.

The comparison between dolomites and limestones reveals distinct conductivity behavior under varying closure stresses and DREC values, highlighting the differential strength responses of these rock types. This signifies that considering such rock-specific behaviors is paramount for effective acid fracturing strategy development.

## Data Availability

The data will be available upon request. The corresponding author Mohammadreza Akbari should be contacted for this purpose.

## Abbreviations

DREC: Dissolved rock equivalent conductivity
RES: Rock embedment strength
NSD: Nasr-El-Din
N&K: Nierode and Kruk
RMSE: Root Mean Squared Error
AARE: Average absolute relative error
XGB: XGBoost method
W_f_: Fracture conductivity
RMSE: Root Mean Square Error
R^2^: Coefficient of determination
AARE: Average Absolute Percentage Error
ANN: Artificial Neural Networks
